# Swiss GPs’ preferences for antidepressant treatment in mild depression: vignette-based quantitative analysis

**DOI:** 10.1186/s12875-021-01621-7

**Published:** 2021-12-30

**Authors:** Michael P. Hengartner, Stefan Neuner-Jehle, Oliver Senn

**Affiliations:** 1 Department of Applied Psychology, Zurich University of Applied Sciences (ZHAW), PO Box 707, CH-8037 Zurich, Switzerland; 2 Institute of Primary Care, University of Zurich and University Hospital Zurich, Zurich, Switzerland

**Keywords:** GPs, Primary Care, Depression, Antidepressants, Prescribing, Treatment Recommendation

## Abstract

**Background:**

GPs frequently prescribe antidepressants in mild depression. The aim of this study was to examine, how often Swiss GPs recommend antidepressants in various clinical presentations of mild depression and which factors contribute to antidepressant treatment recommendations.

**Methods:**

We conducted an online survey among Swiss GPs with within-subject effect analysis. Alternating case vignettes described a typical female case of mild depression according to International Classification of Diseases, 10^th^ edition criteria, with and without anxiety symptoms and sleep problems. GPs indicated for each vignette their preferred treatments (several recommendations were possible). Additionally, we assessed GP characteristics, attitudes towards depression treatments, and elements of clinical decision-making.

**Results:**

Altogether 178 GPs completed the survey. In the initial description of a case with mild depression, 11% (95%-CI: 7%-17%) of GPs recommended antidepressants. If anxiety symptoms were added to the same case, 29% (23%-36%) recommended antidepressants. If sleep problems were mentioned, 47% (40%-55%) recommended antidepressants, and if both sleep problems and anxiety symptoms were mentioned, 63% (56%-70%) recommended antidepressants. Several factors were independently associated with increased odds of recommending antidepressants, specifically more years of practical experience, an advanced training in psychosomatic and psychosocial medicine, self-dispensation, and a higher perceived effectiveness of antidepressants. By contrast, a higher perceived influence of patient characteristics and the use of clinical practice guidelines were associated with reduced odds of recommending antidepressants.

**Conclusions:**

Consistent with depression practice guidelines, Swiss GPs rarely recommended antidepressants in mild depression if no co-indications (i.e., sleep problems and anxiety symptoms) were depicted. However, presence of sleep problems and anxiety symptoms, many years of practical experience, overestimation of antidepressants’ effectiveness, self-dispensation, an advanced training in psychosomatic and psychosocial medicine, and non-use of clinical practice guidelines may independently lead to antidepressant over-prescribing.

**Supplementary Information:**

The online version contains supplementary material available at 10.1186/s12875-021-01621-7.

## Introduction

In Switzerland and most other high-income countries, General practitioners (GPs) treat the majority of patients with depression and also prescribe most antidepressants for depression [1, 2]. However, in the general population and in primary care cohorts, most depression episodes are rated as mild [3, 4]. GPs also frequently ascribe a depression diagnosis even when the liberal diagnostic criteria are not met (also referred to as over-detection or over-diagnosis) [5, 6]. According to the comprehensive meta-analysis by Mitchell et al. [5], the positive predictive value of a GP depression diagnosis was only 42%, indicating that 58% of identified cases were false-positive. Research has further shown that antidepressants are frequently prescribed to these patients with subthreshold depression [5, 6]. It follows that GPs write most of their antidepressant prescriptions for patients with mild depression, including subthreshold cases [1, 6]. According to Martinez et al., British GPs wrote 69% of all antidepressant prescriptions for mild depression, 27% for moderate depression, and only 4% for severe depression [7]. This is a serious issue, for the efficacy of antidepressants has not been firmly established in mild and subthreshold depression [8–10]. By contrast, there is strong evidence that antidepressants convey the risk of various harms [11], including common adverse effects such as sleep problems [12] and sexual dysfunction [13]. The benefit-harm ratio is thus likely unfavourable in many patients with mild depression, which is why most depression practice guidelines, including those published by the National Institute for Health and Care Excellence (NICE), advice against the use of antidepressants as first-line treatment in mild/subthreshold depression [14–16].

The high rate of antidepressant prescribing in mild and subthreshold depression is perceived as “inappropriate” by various mental health professionals and GPs alike [17–21]. Some studies more generally examined why and when GPs prescribe antidepressants, showing that treatment need, i.e., acute distress and illness severity, is among the most important factors [19, 22, 23]. However, it is poorly understood why GPs so frequently prescribe antidepressants specifically in mild depression. According to a recent study, only female gender and having a chronic physical health condition were found to be associated with possible overtreatment with antidepressants when they were not indicated (according to clinical practice guidelines) [21]. However, this study almost exclusively examined patient characteristics. The only GP factor studied was practice location (urban vs. rural).

The aim of this study was thus to examine, which GP and clinical factors may contribute to antidepressant prescribing in mild depression. We hypothesised that anxiety symptoms and sleep problems would be a major driver, as these are co-indications of antidepressant prescribing. We also expected that the GPs’ perceived effectiveness of antidepressants would positively relate to recommending antidepressant treatment.

## Methods

### Ethics and consent

This study was conducted and reported according to the Checklist for Reporting Results of Internet E-Surveys (CHERRIES) [24]. Because this study assessed anonymous data via an online questionnaire, the research ethics committee of the canton of Zurich declared that it was exempt from formal approval according to Swiss law. All participants were informed about the aim of the study (i.e. recognition and treatment of depression in primary care) and that the data were anonymous and protected according to Swiss law. The participants were further informed that the completion of the questionnaire would take about 10 min and a link to the online survey was provided. By agreeing to participate, all respondents gave their informed consent to publish their data.

### Survey development and content

The questionnaire was developed by the study authors. A pilot study was conducted with a paper–pencil version in November 2020 with 12 GPs participating in a quality circle to check the content for clarity and comprehensibility. Based on the GPs’ feedback, a few minor revisions were made to the initial questionnaire and the online survey was programmed. Usability and technical functionality of the electronic questionnaire was tested by the authors. At the beginning of the questionnaire a vignette was presented, describing a married 42-year old female patient with a first episode of mild depression according to the International Classification of Diseases, 10^th^ edition (ICD-10) [25]. In ICD-10, the severity of a depression episode is rated based on both number of symptoms and degree of functional impairment. If no more than five depression symptoms are present and if the patient can fulfill most of his/her daily activities, then the depression episode is considered as mild. In the present study the following four depression symptoms were mentioned: low mood; loss of pleasure, lowered self-esteem, and feelings of worthlessness. It was also stated that the patient was still able to work but felt slightly reduced in her capacity. This indicates a typical case of mild depression episode. The GPs were then asked, which interventions they would recommend. They were presented with the following options and could choose one or several among them: an antidepressant; psychotherapy; watchful waiting; a sedative-hypnotic drug (e.g., a benzodiazepine); phytotherapy (e.g. St. John’s Wort); other intervention.

The GPs were then presented a second vignette, stating that the patient additionally experienced anxiety symptoms, specifically, anxiety towards job loss, divorce, and the future (note that anxiety is not a depression symptom according to ICD-10). The third vignette added that the patient had mild depression with sleep problems (which are listed as a depression symptom), and the last vignette stated that the patient had mild depression with both anxiety symptoms and sleep problems. Thus, in the last vignette the patient had five depression symptoms and good work capacity, which again meets criteria of a mild depression episode (especially in the absence of marked psychomotor retardation and suicidal ideation). The GPs were asked to indicate their preferred treatment options (one or several) as detailed above after each vignette. The exact wording of the vignettes is shown in the online supplement.

The GPs were further asked, to which amount specific mechanisms would underly the remission of non-severe depression by rating their relative contribution (in percentage point) to the total effect. The following options were presented: doctor-patient relationship; pharmacological effect of antidepressants; placebo effect; spontaneous remission; patient characteristics; other factors. The GPs were also surveyed about information and instruments used for clinical decision-making when confronted with a case of depression. They could select among the following options: a diagnostic manual for mental disorders (e.g. DSM-5, ICD-10); questionnaires and symptom checklists (e.g. PHQ-9, BDI); clinical practice guideline (e.g. guideline from the Swiss psychiatric association, NICE); another instrument; no instrument (clinical impression). Finally, in the last section the GPs answered questions about their socio-demographics, their professional background and education, and their work environment. These questions also included drug self-dispensation, which in Switzerland indicates that a GP runs his/her own pharmacy. The questionnaire comprised altogether 21 items.

### Survey administration and quality checks

The response options to the four vignettes were alternated in sequence, otherwise no randomisation or alternation of items was applied. Two completeness checks were applied. First, a response to each vignette was mandatory to proceed. Second, the perceived effect of the main mechanism underlying the remission of depression had to amount to 100% in total, otherwise proceeding to the next item was not possible. All other items had no completeness checks and could be omitted or skipped. Respondents were able to review and change their answers. Only one entry was allowed per IP address to prevent multiple entries from the same individual. No other techniques were applied to prevent multiple entries. Inspection of the data revealed no peculiar response patterns.

### Recruitment and administration

The sample for this closed survey was recruited via a mailing list consisting of GPs affiliated to the Institute of Primary Care at the University of Zurich. Potential participants were members of the FIRE project (*n* = 480), a GP research network in different German-speaking parts of Switzerland [26], respondents of a previous outreach among all GPs in the Canton of Zurich who had indicated that they were generally interested in participating in surveys (*n* = 96), and GPs involved in undergraduate and postgraduate medical training (*n* = 300). The letter advertising the questionnaire is shown in the supplement. The electronic questionnaire was posted on the online survey platform UniPark.com and was accessible only via the link provided in the information letter. No incentives were offered. Data were collected in March 2021. A reminder to participate in the study was sent out two weeks after the first advertisement.

### Statistical analysis

The main analysis was conducted with a series of Generalised Estimating Equations (GEE) [27]. GEE models were introduced to fit regression analyses that account for within-subject correlation, which is an inherent part in studies that rely on repeated outcome measures. The treatment recommendations given after each vignette were entered successively as the outcome variable, and the vignette (i.e., main description of mild depression; mild depression with anxiety symptoms; mild depression with sleep problems; mild depression with both sleep problems and anxiety) as the predictor variable. We fitted binomial models with logit link-function and the within-subject covariance was specified with the “unstructured” correlation type to avoid having any constraints on the covariance structure. Marginal means with 95% confidence intervals were estimated to quantify the rate of recommendations for each treatment based on the case vignette. All analyses were conducted with SPSS version 24 for Windows.

## Results

The information letter with the link to the online questionnaire was send to 876 physicians. Some of these were pediatricians and some doctors informed us that they were already retired, so the eligible sample was necessarily smaller, but the exact number is unknown. Altogether 178 GPs completed the online questionnaire, producing a response rate of > 20%. The participants had a mean age of 52.2 years and a mean practical experience of 18.0 years. A small majority was male (56.5%) and most worked in group practice (82.5%) and in an urban/suburban environment (79.6%). More details are provided in Table 1.

**Table 1:** Sample characteristics (*n* = 178)

	Minimum / Maximum	Mean (SD)
Age	33 years / 72 years	52.2 (9.0)
Practical experience	1 year / 40 years	18.0 (9.8)
		*N* (valid percent)
Sex		
	Female	77 (43.5%)
	Male	100 (56.5%)
	Missing	1
Work location		
	Urban	79 (44.6%)
	Suburban	62 (35.0%)
	Rural	36 (20.3%)
	Missing	1
Work setting		
	Single practice	30 (16.9%)
	Group practice	146 (82.5%)
	Hospital	1 (0.6%)
	Missing	1
Specialty		
	General internal medicine	166 (94.3%)
	Family medicine	10 (5.7%)
	Missing	2
Advanced training		
	Psychosomatic and psychosocial medicine	18 (10.1%)
	Delegated psychotherapy	16 (9.0%)
	None of the above	144 (80.9%)
Attendance of quality		
circles per year	No attendance	8 (4.5%)
	1–5 times	20 (11.2%)
	6–10 times	77 (43.3%)
	11–15 times	28 (15.7%)
	16–20 times	17 (9.6%)
	> 20 times	28 (15.7%)
Self-dispensation		
	Yes	139 (79.0%)
	No	37 (21.0%)
	Missing	2

**Fig. 1:**
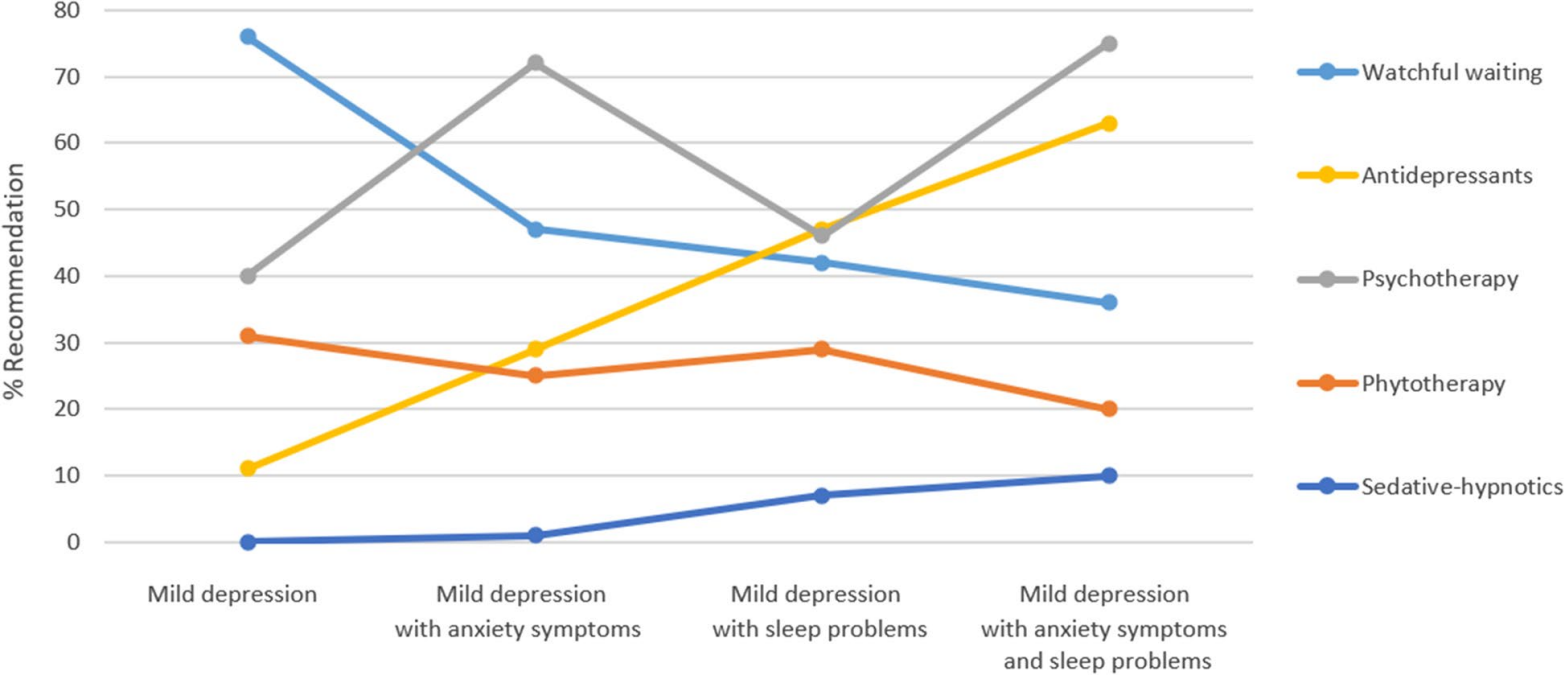
Rate of treatment recommendations based on case description (vignette)

The treatment recommendations according to case description (vignette) are shown in Table 2. For the initial case description of mild depression, the most common treatment recommendation was watchful waiting (75.8%). If anxiety symptoms were added, doctors most often recommended psychotherapy (72.5%). When mild depression with sleep problems were depicted in the vignette, doctors most frequently recommended antidepressants (47.2%), psychotherapy (45.5%), or watchful waiting (42.1%). If a case with mild depression and both anxiety symptoms and sleep problems was depicted, then doctors most often recommended psychotherapy (75.3%) and antidepressants (63.5%). The change in treatment recommendations based on case description is also shown in Fig. 1. Estimates of the grand marginal mean (average rate of recommendations across vignettes) were 59% (95%-CI: 53%—65%) for psychotherapy, 51% (45%—57%) for watchful waiting, 35% (29%—41%) for antidepressants, and 26% (22%—31%) for phytotherapy. The combination of antidepressants and psychotherapy was recommended by 22% (18%-28%) of GPs across vignettes (7% in mild depression; 22% in mild depression with anxiety symptoms; 24% in mild depression with sleep problems; and 50% in mild depression with both anxiety symptoms and sleep problems). The GEE model for sedative-hypnotic drugs did not converge due to small numbers.

**Table 2:** Percent of doctors recommending a specific treatment according to case description. Several recommendations were possible

Recommendation	Vignette			
	Mild depression	Mild depression with anxiety symptoms	Mild depression with sleep problems	Mild depression with anxiety symptoms and sleep problems
	Mean (95%-CI)	Mean (95%-CI)	Mean (95%-CI)	Mean (95%-CI)
Watchful waiting	76% (69%—82%)	47 (39%—54%)	42% (35%—50%)	36% (29%—43%)
Antidepressant	11% (7%—17%)	29% (23%—36%)	47% (40%—55%)	63% (56%—70%)
Psychotherapy	40% (33%—47%)	72% (65%—79%)	46% (38%—53%)	75% (68%—81%)
Phytotherapy (e.g. St John’s wort)	31% (25%—39%)	25% (19%—32%)	29% (22%—36%)	20% (14%—26%)
Sedative-hypnotic drug (e.g. benzodiazepine)	0%^a^	1%^a^	7%^a^	10%^a^

^a^Confidence interval could not be estimated because the GEE model did not converge

The results for the relative contribution of specific mechanisms to the remission of non-severe depression are shown in Table 3. The doctor-patient relationship (median effect: 31%) was rated most important, followed by antidepressants’ pharmacological effect (20%) and patient characteristics (20%), spontaneous remission (13%), and the placebo effect (12%). Wilcoxon signed rank tests showed that the doctor-patient relationship was rated more important than any other mechanism (all *p* < 0.001). Both antidepressants’ pharmacological effect and patient characteristics were rated more important than spontaneous remission and placebo effect (both *p* < 0.001).

**Table 3:** GPs estimates of the relative effect of specific mechanisms underlying the remission of non-severe depression

Mechanism	Median	Interquartile range
Antidepressants’ pharmacological action	20%	15%—30%
Doctor-patient relationship (therapeutic alliance)	31%	25%—50%
Placebo effect	12%	7%—20%
Spontaneous remission	13%	6%—24%
Patient characteristics	20%	10%—34%

Finally, the GPs indicated that they would rarely use a specific instrument to aid diagnosis and clinical decision-making in depression. Altogether, 17.4% responded that they would use a diagnostic manual for mental disorders (e.g., DSM-5, ICD-10), 21.3% would use a depression questionnaire (e.g., PHQ-9, BDI), and 15.2% would adhere to a clinical practice guideline (e.g., guideline by the Swiss psychiatric association, NICE). The majority of GPs, i.e., 65.7%, indicated that they would not use any instrument but fully rely on their clinical impression.

In the univariable GEE model, the following variables showed some association (*p *< 0.1) with recommending antidepressant treatment: GPs older age (*p* = 0.002), more years of practical experience (*p* = 0.001), urban/suburban work location (*p* = 0.084), advanced training in psychosomatic and psychosocial medicine (*p* = 0.037), self-dispensation (*p* = 0.036), higher perceived effect of antidepressants’ pharmacological action (*p* = 0.049), lower perceived effect of spontaneous remission (*p* = 0.037), lower perceived effect of patient characteristics (*p* = 0.003), and non-use of clinical practice guidelines (*p* = 0.004). Age and years of practical experience as well as perceived effect of spontaneous remission and perceived effect of patient characteristics were highly intercorrelated (both r > 0.6). To avoid multicollinearity bias, only the stronger predictor of these pairs was selected, i.e., years of practical experience and perceived effect of patient characteristics. The final multivariable predictor model is shown in Table 4.

**Table 4:** Multivariable predictors of antidepressant treatment recommendation in mild depression

Predictor	Indicator	*OR* (95%-CI)	*p*
Vignette			
	With anxiety and sleep problems (*n* = 169)	20.0 (11.5–35.2)	< 0.001
	With sleep problems (*n* = 169)	8.8 (5.2–14.9)	< 0.001
	With anxiety symptoms (*n* = 169)	3.6 (2.3–5.6)	< 0.001
	Mild depression (*n* = 169)	Reference	
Years of practical experience			
	1 SD increase (*n* = 169)	1.4 (1.1–1.9)	0.009
Work location			
	Rural (*n* = 35)	2.1 (1.0–4.4)	0.061
	Suburban (*n* = 62)	1.4 (0.8–2.5)	0.264
	Urban (*n* = 72)	Reference	
Advanced training in psychosomatic and psychosocial medicine			
	Yes (*n* = 15)	4.1 (1.6–10.4)	0.003
	No (*n* = 154)	Reference	
Self-dispensation			
	Yes (*n* = 133)	2.3 (1.2–4.6)	0.017
	No (*n* = 36)	Reference	
Perceived effect of antidepressants’ pharmacological action			
	1 SD increase (*n* = 169)	1.4 (1.1–1.8)	0.005
Perceived effect of patient characteristics			
	1 SD increase (*n* = 169)	0.8 (0.6–1.0)	0.040
Use of clinical practice guidelines			
	Yes (*n* = 25)	0.3 (0.1–0.6)	< 0.001
	No (*n* = 144)	Reference	

Independent of the case description (vignette) and all other predictor variables, an advanced training in psychosomatic and psychosocial medicine (*OR* = 4.1) strongly increased the odds of recommending antidepressants, while using clinical practice guidelines (*OR* = 0.3) strongly reduced the odds. Self-dispensation (*OR* = 2.3) as well as one standard deviation higher scores on both years of practical experience (*OR* = 1.4) and perceived effect of antidepressants’ pharmacological action (*OR* = 1.4) also increased the odds of recommending antidepressant treatment, while one standard deviation higher scores on perceived effect of patient characteristics (*OR* = 0.8) was associated with a slightly lower odds of recommending antidepressant treatment.

If GP age and perceived effect of spontaneous remission were entered into the model instead of years of practical experience and perceived effect of patient characteristics, one standard deviation higher age (*OR* = 1.4, 95%-*CI* = 1.1–1.8, *p* = 0.010) was associated with an increased odds of recommending antidepressants, whereas one standard deviation higher perceived effect of spontaneous remission was non-significantly associated with a reduced odds (*OR* = 0.8, 95%-*CI* = 0.6–1.0, *p* = 0.058). The associations of the other predictor variables remained virtually identical to the results reported in Table 4.

The multivariable predictor model for recommending watchful waiting and other analyses are shown in the online supplement.

## Discussion

Consistent with depression treatment guidelines [14–16], Swiss GPs mostly recommended psychotherapy (59%) and watchful waiting (51%) for different clinical presentations of a first episode of mild depression. Antidepressants were significantly less frequently recommended (35%). However, the recommendation of antidepressant treatment strongly varied based on the specific symptoms depicted: in the initial description of a case with mild depression, antidepressants were rarely recommended (11%), but if sleep problems were added to the vignette, then about half of GPs recommended antidepressants (47%). If both sleep problems and anxiety symptoms were mentioned, then almost two-thirds of GPs recommended antidepressants (63%). Various factors as discussed below were independently associated with GPs’ preferences for antidepressant treatment.

The rate of 35% for recommending antidepressant treatment across case descriptions of mild depression (with and without sleep problems and anxiety) is consistent with an actual prescription rate of 34% by German GPs when they issued a diagnosis of mild depression according to a large German primary care study [28]. In our study, antidepressants were more frequently recommended if sleep problems and, especially, if both sleep problems and anxiety symptoms were depicted in the case vignette. This indicates that GPs strongly base their treatment recommendations on the presence of specific symptoms. For example, the antidepressant drugs trazodone and mirtazapine have a modest effect on insomnia [29, 30], which might have triggered the GPs’ decision to recommend antidepressant treatment in cases with additional sleep problems. Similarly, the higher rate of recommending antidepressants in the case of additional anxiety symptoms may be explained by the anxiolytic effect of various antidepressant drugs [31, 32]. In fact, it appears that antidepressants are more effective in anxiety symptoms than in depression symptoms. According a large pragmatic effectiveness trial in primary care patients with depression, antidepressants barely improved depression symptoms, but they were more effective in alleviating anxiety symptoms [33].

The presence of sleep problems and anxiety symptoms may also suggest higher treatment need, a factor known to influence clinical decision-making in depression [19, 22, 23]. Although in each vignette the case description was consistent with a mild depression episode according to ICD-10 criteria (no more than five depression symptoms and capacity to work), it is likely that some GPs judged the presence of both anxiety symptoms and sleep problems to indicate a more severe disorder. Given that there is uncertainty on how the severity of depression should be assessed, and how well the number of symptoms correlates with the clinical global impression, this is an issue that warrants further research [34].

Noteworthy, in our study the GPs did not recommend sedative-hypnotic drugs in the initial mild depression vignette (0%), and rarely when sleep problems (7%) and both anxiety symptoms and sleep problems (10%) were depicted. Across vignettes, 20% to 31% of Swiss GPs also recommended phytotherapy, which is consistent with the literature showing that St John’s Wort is a valuable treatment option in non-severe depression, as it is as effective as antidepressants but better tolerated [35, 36].

The doctor-patient relationship (therapeutic alliance) was attributed the most importance in the remission of depression, which is consistent with the strong preference for watchful waiting in the original case vignette of mild depression without anxiety symptoms and sleep problems. Based on process-outcome research in psychotherapy, there is indeed strong evidence to assume that a good doctor-patient relationship is probably the single most important treatment factor in depression [37, 38].

We found that a higher perception of antidepressant effectiveness was associated with recommending antidepressant treatment more often. Noteworthy is that, on average, GPs attributed 20% of observed improvements in depression to antidepressants’ pharmacological effects. This is broadly in line with comprehensive reviews and meta-analyses of antidepressant efficacy trials, which indicate that only 10–20% of the treatment outcome in depression is due to antidepressants’ pharmacological effects [39, 40]. This finding underscores the importance of an evidence-based approach to clinical decision-making, given that GPs who overestimate the effectiveness of antidepressants more frequently (and perhaps inappropriately) recommend antidepressant treatment. Likewise, more years of practical experience may also contribute to potential over-prescribing in mild depression, since repeated clinical observations of improvements after initiation of antidepressant treatment are possibly misattributed to the drugs’ pharmacological effects rather than to spontaneous remission and non-pharmacological treatment factors such as the doctor-patient relationship [17, 41].

The use of clinical practice guidelines was associated with reduced odds of recommending antidepressants for mild depression. This finding is consistent with the prevailing advice to not use antidepressants as first-line treatment in mild depression but watchful waiting and/or low-intensity psychosocial interventions instead [14–16]. Unfortunately, only 15% of GPs indicated that they would consult depression practice guidelines when confronted with a case of depression. Increasing awareness of and adherence to clinical practice guidelines for depression may thus curb inappropriate and potentially harmful antidepressant prescribing in mild depression [17, 18].

Finally, we found that GPs who run their own pharmacy (self-dispensation) more frequently recommended antidepressants. This suggests that financial incentives may play a role, as GPs can make an additional income by prescribing more drug. However, there is no consistent support for this assumption, for the scientific evidence on the effect of self-dispensation is mixed [42, 43]. Nevertheless, it is worthy of note that according to a large survey among US physicians, 71% believed that physicians provide unnecessary procedures when they profit from them [44]. More research into a possible link between self-dispensation and overprescribing and its underlying mechanism is therefore required. We also found that an advanced training in psychosomatic and psychosocial medicine was associated with an increased odds of recommending antidepressants. This training stresses the links between mind and body and the parity between mental and physical health. Therefore, it might increase awareness of depression and make GPs more inclined to prescribe antidepressants, especially when somatic symptoms such as sleep problems, fatigue, and lack of energy are present. However, the training also stresses the importance of psychosocial interventions in both mental and physical conditions. The strong association between advanced training in psychosomatic and psychosocial medicine and a GP preference for antidepressant treatment is therefore a surprising finding for which we found no support in the scientific literature [28]. As only 18 GPs participating in this survey had completed this advanced training, we suggest it could also be a methodological artefact and thus requires replication in future studies with larger samples.

### Strengths and limitations

The alteration of vignettes and a quantitative analysis of within-subject effects allowed to provide novel insights into potential factors related to antidepressant prescribing in mild depression. To the best of our knowledge, this is the first study examining the impact of different clinical presentations of depression. However, this study also has some limitations. First and foremost, the data was based on a survey and GPs only indicated their preferred treatments. As social desirability may play a role, these answers may deviate from their actual treatment recommendations in daily practice. In addition, treatment recommendations do not necessarily reflect true prescribing behaviour, since the role of the patients, especially their treatment preferences, were not taken into account. Likewise, patient autonomy and the decision-making process were not addressed and may also influence actual prescribing behaviour in daily practice. The vignette described the case of a married 42-years old woman, thus generalisation to other cases might be limited. In a man of other socio-economic background (e.g., young, single, and unemployed), the treatment recommendations might differ. The response rate was approximately 20%, so the external validity of the study findings may be limited if survey respondents were not representative of the German-speaking Swiss GP population. While GP mean age, work location and setting closely matched the characteristics of the GPs included in the FIRE database and the Swiss GP population, with 44% female GPs were overrepresented in this study (women account for only 35% of the FIRE database and 36% of the Swiss GP workforce) [45]. Finally, it is also possible that the questionnaire was mostly completed by GPs with a special interest in depression, which may introduce bias.

## Conclusions

We conclude that the treatments recommended by Swiss GPs for a typical case of mild depression are mostly in line with established practice guidelines, strongly favouring watchful waiting over antidepressant treatment. However, if both sleep problems and anxiety symptoms are present, then GPs seem to prefer antidepressant treatment over watchful waiting. The clinical presentation in mild depression thus plays an important role. Educating GPs that, independent of treatment administered, mild depression has typically a good prognosis [46–48], and that watchful waiting is as effective as antidepressant treatment [10, 49], may curb antidepressant over-prescribing in mild depression. We further showed that GPs who overestimate the effectiveness of antidepressants more often recommend antidepressant treatment. A critical, evidence-based evaluation of the efficacy and safety of antidepressants is thus important in medical education and practice. Finally, given that only 15% of GPs made use of depression practice guidelines, more adherence to these guidelines, which advise watchful waiting as first-line treatment in mild depression, may further restrict inappropriate antidepressant prescribing [17, 18].

## Supplementary Information

**Additional file 1.**

## Data Availability

The raw data and statistical code can be obtained from the first author.
