# Correction to: Swiss GPs’ preferences for antidepressant treatment in mild depression: vignette-based quantitative analysis

**DOI:** 10.1186/s12875-022-01626-w

**Published:** 2022-02-08

**Authors:** Michael P. Hengartner, Stefan Neuner-Jehle, Oliver Senn

**Affiliations:** 1 Department of Applied Psychology, Zurich University of Applied Sciences (ZHAW), PO Box 707, CH-8037 Zurich, Switzerland; 2 Institute of Primary Care, University of Zurich and University Hospital Zurich, Zurich, Switzerland

**Correction to: BMC Family Practice 22, 261 (2021)**

10.1186/s12875-021-01621-7

In the original publication of this article [1], there was an error in Fig. 1. The legend for the categories on the X-axis were misaligned. The correct figure is given below. The original article has been corrected.

**Fig. 1:**
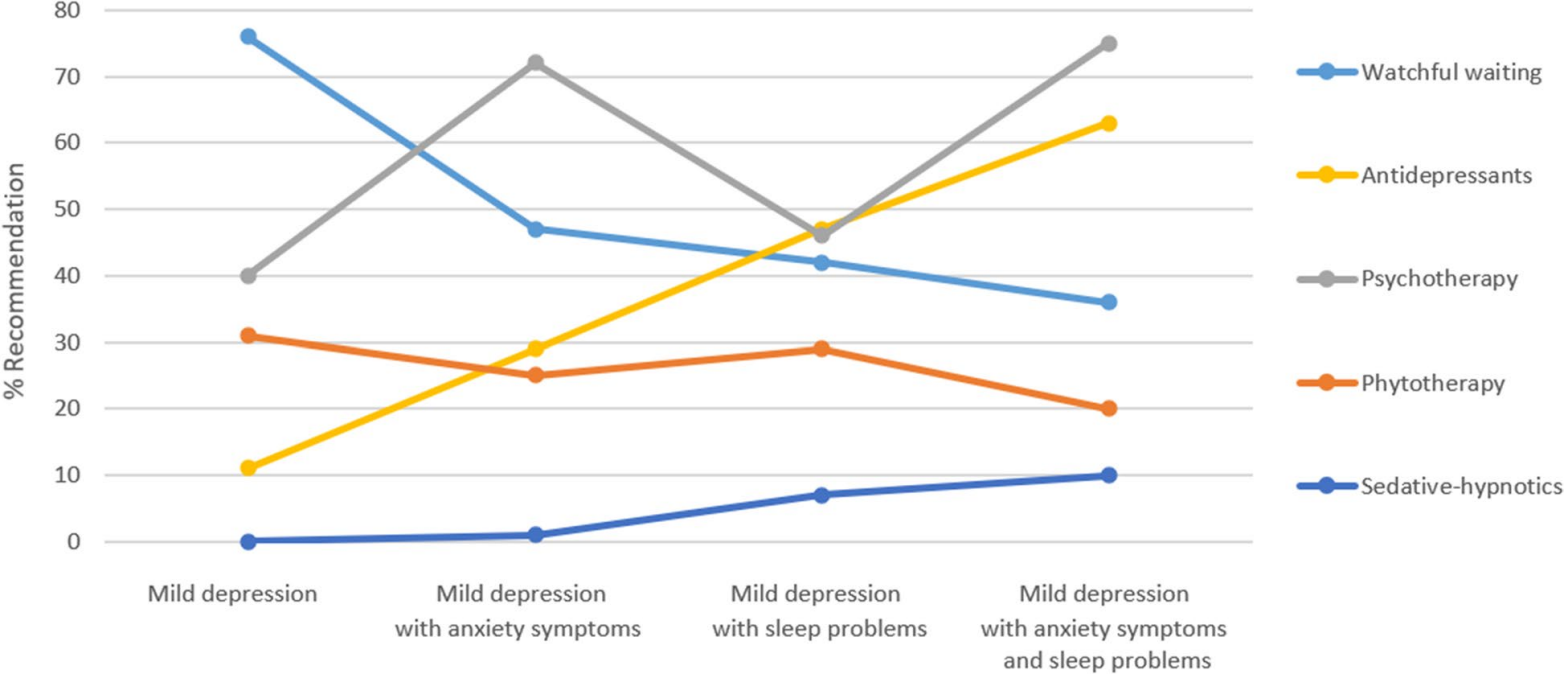
Rate of treatment recommendations based on case description (vignette)
