# Supplementary material for: Swiss GPs’ preferences for antidepressant treatment in mild depression: vignette-based quantitative analysis
Source: BMC Fam Pract. 2021 Dec 30;22:261. doi: 10.1186/s12875-021-01621-7 (PMC8717647; doi:10.1186/s12875-021-01621-7)
Supplement: Supplementary file 1 — Additional file 1. [file 12875_2021_1621_MOESM1_ESM.docx]

**SUPPLEMENTARY MATERIAL**

**Swiss GPs’ preferences for antidepressant treatment in mild depression: vignette-based quantitative analysis**

Michael P. Hengartner^1^, Stefan Neuner-Jehle^2^, Oliver Senn^2^

^1^ Department of Applied Psychology, Zurich University of Applied Sciences, Switzerland

^2^ Institute of Primary Care, University of Zurich and University Hospital Zurich, Switzerland

Corresponding author:

Michael P. Hengartner, PhD

Department of Applied Psychology

Zurich University of Applied Sciences (ZHAW)

PO Box 707

CH-8037 Zurich, Switzerland

[heng@zhaw.ch](mailto:heng@zhaw.ch)

Tel: +41589348382

**METHODS**

**Vignette**

Case description of mild depression:

“A 42 years old woman reports having low mood and loss of pleasure for the first time. Additionally, she indicates lowered self-esteem and feelings of worthlessness. The symptoms would last for about three to four weeks. The woman is still able to work, but she feels slightly reduced in her capacity. Pre-existing somatic and mental disorders are not known. A physical examination and laboratory tests are normal. The social anamnesis revealed that the women has repeated marital conflicts with her husband and that she is increasingly unhappy at her workplace”.

Case description of mild depression with generalised anxiety symptoms:

“If the patient, during the same first consultation, in addition to the symptoms reported in the main vignette, also indicates various anxieties (anxiety towards job loss, anxiety towards divorce, anxiety towards the future), which intervention would you recommend then?”

Case description of mild depression with sleep problems:

“If the patient, during the same first consultation, in addition to the symptoms reported in the main vignette, also indicates sleep problems (insomnia early and in the middle of the night), which intervention would you recommend then?”

Case description of mild depression with both generalised anxiety symptoms and sleep problems:

“If the patient, during the same first consultation, in addition to the symptoms reported in the main vignette, also indicates both various anxieties and sleep problems, which intervention would you recommend then?”

**Information letter**

The online survey was advertised with the following information letter: “Primary care practices are usually the first contact point for patients with depression symptoms. As a GP you play a major role in both the timely detection of depression and the treatment of these patients. Although depression is a frequent medical condition, GPs care of depression patients and possible barriers to treatment are poorly studied. Therefore, the Institute of Primary Care of the University of Zurich in collaboration with the Psychological Institute of the Zurich University of Applied Sciences conduct a survey on the treatment of depression in primary care practices”. The principal investigator of the study (MPH) and his contact information were also mentioned.

**RESULTS**

**Additional findings**

GPs rated the efficacy of antidepressants in relation to both psychotherapy and benzodiazepines. To do so they had to move a slider on a 11-point scale, where 1 denoted antidepressants being clearly superior, 6 equally effective, and 11 the other treatment (i.e. psychotherapy or benzodiazepines) being clearly superior. They were also asked whether they thought that the effectiveness of psychotherapy is age-related and whether psychotherapy is accessible.

The GPs did not consider antidepressants more effective than psychotherapy in non-severe depression. The median score was 7 (interquartile range: 6-9), thus slightly in favour of psychotherapy. When asked whether the effectiveness of psychotherapy, relative to antidepressants, is different in people aged >75 years, 32.8% of GPs indicated that the effectiveness of psychotherapy decreases relative to antidepressants, 55.4% stated there is no age-related effect, and 11.9% indicated to the effectiveness of psychotherapy increases relative to antidepressants. The accessibility of psychotherapy was rated as follows: very poor (no therapists available, long waiting list): 17.4%; rather poor (few therapists available; rather long waiting list): 57.9%; neither poor nor good: 10.7%; rather good (some therapists available; rather short waiting list): 12.4%; very good (many therapists available, short waiting list): 1.7%.

Comparing the effectiveness of antidepressants to benzodiazepines (1: antidepressants clearly superior; 6: both treatments equally effective; 11: benzodiazepines clearly superior) revealed a median score of 3 (2-4), thus strongly favouring the effectiveness of antidepressants.

Given that watchful waiting is widely accepted to be the first-line intervention in patients with mild depression, we re-ran the multivariable prediction model detailed in the main text for recommending watchful waiting as the outcome variable. Independent of the case description (vignette), a specialization in family medicine as compared to general internal medicine (p=0.066), the perceived effect of antidepressants’ pharmacological action (p=0.028), the perceived effect of spontaneous remission (p=0.057), the perceived effect of the doctor-patient relationship (p=0.057), and lacking acceptance of treatment recommendations (p=0.015) showed some association. The perceived effects of spontaneous remission and doctor-patient relationship were strongly correlated (r=0.53), therefore we included only the former in the multivariable GEE model to avoid multicollinearity bias. The results are shown in the supplementary Table 1. Lacking acceptance of treatment recommendations (OR=0.6) and one standard deviation lower scores on the perceived effect of antidepressants’ pharmacological action (OR=0.7) were associated with a significantly lower odds of recommending watchful waiting, while one standard deviation higher scores on the perceived effect on spontaneous remission was associated with a significantly higher odds (OR=1.3).

Supplementary Table 1: Multivariable predictors of recommending watchful waiting

| Predictor | Indicator | OR (95%-CI) | p |
| --- | --- | --- | --- |
| Vignette | With anxiety and sleep problems  With sleeps problems  With anxiety symptoms  Mild depression | 0.2 (0.1-0.2)  0.2 (0.1-0.3)  0.3 (0.2-0.4)  Reference | <0.001  <0.001  <0.001 |
| Specialty | Family medicine  General internal medicine | 0.4 (0.1-1.2)  Reference | 0.086 |
| Lacking acceptance of treatment recommendations | Yes  No | 0.6 (0.4-1.0)  Reference | 0.049 |
| Perceived effect of antidepressants’ pharmacological action | 1 SD increase | 0.7 (0.5-0.9) | 0.008 |
| Perceived effect of spontaneous remission | 1 SD increase | 1.3 (1.0-1.7) | 0.043 |
